# ‘Do I know you?’ Categorizing individuals on the basis of familiarity in kea (*Nestor notabilis*)

**DOI:** 10.1098/rsos.230228

**Published:** 2023-06-21

**Authors:** Elisabeth Suwandschieff, Roger Mundry, Kristina Kull, Lena Kreuzer, Raoul Schwing

**Affiliations:** ^1^ Research Station Haidlhof, Comparative Cognition, Messerli Research Institute, University of Veterinary Medicine, Vienna, Austria; ^2^ Platform Bioinformatics and Biostatistics, University of Veterinary Medicine, Vienna, Austria; ^3^ Cognitive Ethology Laboratory, German Primate Center, Leibniz Institute for Primate Research, Göttingen, Germany; ^4^ Department for Primate Cognition, Georg-August-University Göttingen, Göttingen, Germany; ^5^ Leibniz-ScienceCampus Primate Cognition, Göttingen, Germany; ^6^ Division of Livestock Sciences, Department of Sustainable Agricultural Systems, University of Natural Resources and Life Sciences, Vienna, Austria

**Keywords:** kea, Nestor notabilis, avian cognition, conceptual familiarity, two-choice discrimination task

## Abstract

Categorizing individuals on the basis of familiarity is an adaptive way of dealing with the complexity of the social environment. It requires the use of conceptual familiarity and is considered higher order learning. Although, it is common among many species, ecological need might require and facilitate individual differentiation among heterospecifics. This may be true for laboratory populations just as much as for domesticated species and those that live in urban contexts. However, with the exception of a few studies, populations of laboratory animals have generally been given less attention. The study at hand, therefore, addressed the question whether a laboratory population of kea parrots (*Nestor notabilis*) were able to apply the concept of familiarity to differentiate between human faces in a two-choice discrimination task on the touchscreen. The results illustrated that the laboratory population of kea were indeed able to differentiate between familiar and unfamiliar human faces in a two-choice discrimination task. The results provide novel empirical evidence on abstract categorization capacities in parrots while at the same time providing further evidence of representational insight in kea.

## 1. Introduction

The ability to differentiate between individuals can be of great importance for survival, as it allows animals to make more informed decisions on interacting with surrounding animals [1]. Research into this has shown that many species have the ability to recognize individual conspecifics [2–5], and that this forms the basis of their social network. At its basic level, individuals accomplish the task of discrimination by integrating different sensory channels and relying on environmental and physical information such as shape, size, odour, colour and sound [6,7]. Different species have developed different competences—with regard to the information they process—according to their physical characteristics, social systems, habitat and ecological niche. For instance, humans exhibit a predisposition for visual cues with a distinct attentional bias towards faces [7,8].

Although thought to be a predominantly human trait, studies have shown that humans share this bias with other species that live in tight social networks, with a heavy reliance on visual information [7]. Wasps (both *Polistes fuscatus* and *Liostenogaster flavolineata*), which were previously believed to be mainly reliant on chemical information, have illustrated a bias towards facial features when identifying nest mates [9–12]. Along with *Hymenoptera*, non-human primates *(Macaca mulatta* and *Pan troglodytes)* [13–15], birds (Galliformes and Psittaciformes) [16–18], Artiodactyla such as sheep (*Ovis aries*) [19–21] and cows (*Bos taurus*) [22] and Cichlidae [23,24] have been shown to rely on facial features as visual recognition cues. Furthermore, Marzluff *et al.*'s [25] study on wild American crows (*Corvus brachyrhynchos*) indicated that human facial features play an important role in heterospecific recognition. And Stephan and colleagues illustrated in their 2012 study that ‘facial information alone is sufficient for pigeons (*Columba livia*) to discriminate among humans’ [26, p. 78].

Categorizing conspecifics on the basis of familiarity is an adaptive way of dealing with the complexity of the social environment. Categorization of conspecifics requires the use of conceptual familiarity, which goes beyond mere perceptual features and is considered higher order learning [27]. As a ‘fundamental cognitive process' [28, p. 983] it entails the reduction of complexity through identifying and relying on shared object properties. In other words, an individual can process that other individuals of the same category share many uniform or similar properties and can therefore be identified swiftly. Pre-experience is at the core of this ability, which allows to group familiar individuals into clusters or classes and helps differentiate between those who belong to this class and those who do not [28].

Most research concerning conceptual familiarity has focused on conspecifics, ecological need might, however, necessitate and consequently facilitate individual differentiation among heterospecifics [26]. Applying familiarity as a discriminative feature to heterospecific discrimination is very useful, not only for predator avoidance but also for animals in regular contact with human beings. Pre-experience and pre-exposure facilitate the application of heterospecific differentiation for those species that would not apply or develop this ability in their natural environment. Laboratory animals, like animals living in urban contexts, benefit from flexibly adapting to the increased anthropogenic disturbance [29] they face in the laboratory environment, and hence the development of the ability to differentiate between heterospecifics should be facilitated. This is especially true for those species with demonstrated ‘high cognitive abilities’ [25] and that live in complex social structures such as, for instance, kea parrots (*Nestor notabilis*). In line with the ‘pre-exposure hypothesis' (postulated by Lee and colleagues [30]), domesticated species and those living in urban environments have frequently been tested [21,25,26,29–34]. However, populations of laboratory animals have generally been ignored with the noteworthy exceptions of the pigeon studies [26,28] and the most recent study by Leinwand *et al*. [35] of a zoo ape population (chimpanzees (*Pan troglodytes*) and gorillas (*Gorilla gorilla gorilla*)).

In their 2012 study ‘Have we met before? Pigeons recognize familiar human faces’ Stephan and colleagues illustrated that pigeons were able to discriminate between familiar and unfamiliar human facial features. Stephan *et al.* tested whether pigeons were able to transfer the ‘discriminative rule of familiarity’ [26, p. 79] acquired in an object training task, to a novel set of stimuli, namely human faces. The results showed that four out of eight test group birds successfully categorized individual heterospecifics on the basis of pictorial representations and that none of the control birds succeeded in this task—indicating that pigeons were indeed able to transfer the concept of familiarity to heterospecifics. The 2012 and 2013 studies [26,28] showed that pigeons were able to use the concept of familiarity as a categorical rule to classify between familiar and unfamiliar humans as well as objects. However, as mentioned before, with the exception of the pigeon studies very little is known about this ability in other laboratory animals.

Examining the ability to discriminate among both con- and hetero-specifics often entails the provision of pictorial or two-dimensional representations of the actual referents. Using pictures can be advantageous in two main ways. First, it allows the direct selection and manipulation of certain features and second, the restriction or exclusion of others [26]. An example of this would be selecting only facial features while removing all other information such as body, clothing and background. This process ensures that only the actual features of interest are being tested and no other background information can be used to solve the discrimination task. However, being able to correctly identify two-dimensional representations (i.e. photographs) of real-life referents (such as objects, hetero- or con-specifics) presents a high level of abstraction, as it requires dual representation ([36] in [27], p. 109). Hence, the animals are required to understand that the two-dimensional representations are references to real-life objects or beings. So far, mainly mammals and pigeons have been shown to possess representational insight [27] as described above. However, touchscreen experiments have shown that also kea parrots can successfully apply picture-to-object recognition, revealing their ability to infer the connection between real-life referents and their two-dimensional representations [37].

Kea are a large parrot species endemic to the alpine environments (Southern Alps of New Zealand), that live in fission-fusion social flocks and are extractive foragers. They faced very little predation in their natural environment, which together with their seasonal feeding strategies has rendered them highly neophilic [38]. Their complex social structure along with their highly inquisitive nature [39] suggests that they are predisposed to behavioural flexibility. Kea have well-marked technical intelligence [40], illustrated representational insight [37] and are exceptional problem solvers [38,40–42]. We know that they have the ability to successfully solve discrimination tasks [37,43], suggesting that individual differentiation may also be in their cognitive repertoire. Although kea may not need the ability to differentiate between heterospecifics in their natural environment, considering their high cognitive abilities they may be able to adapt more quickly to environments that necessitate this ability. This would be in line with the predictions of Lee *et al*. [30] who postulated that ‘higher cognitive abilities may hypothetically pre-adapt birds to be more susceptible to the pre-exposure effect, leading to more pronounced (faster) development of abilities to recognize individual humans in birds of higher cognitive/perceptual abilities' [30, p. 823]. Considering the above profile, it seems reasonable to assume that kea living in laboratory conditions would not only benefit from heterospecific recognition, but very likely have acquired this ability accordingly.

The kea at the research station Haidlhof have interacted with humans all their lives. Anecdotally it seems clear that they can indeed differentiate between human beings. However, formal investigation into this field is still missing, not only for kea but indeed for the entire parrot taxa. Furthermore, very little is known about the ability of kea to process human facial information, which has never been tested before. Tying together the kea's ability to solve discrimination tasks with their capacity for representational insight, the study at hand investigated whether kea are able to apply familiarity as a discriminative feature to heterospecific discrimination. The objective of the study was, therefore, to verify whether kea living in a laboratory setting could differentiate between familiar and unfamiliar heterospecifics using facial information alone. To this end, we had two research questions to address. First, can kea recognize human faces by using pictorial or two-dimensional representations on a touchscreen? We hypothesized that differences in facial features will affect the kea's ability to choose in a two-choice task and the prediction that kea will be able to recognize and discriminate on the basis of pictures of different human faces. The second research question addressed the question whether kea can use familiarity to categorize different human faces? We hypothesized that different levels of familiarity will affect kea's ability to discriminate between familiar and unfamiliar human faces. We predicted that kea will be able to use familiarity to categorize familiar and unfamiliar human faces, and outperform a control group that cannot use such categories when solving the task.

## 2. Methods

The methods for testing whether kea can discriminate between familiar and unfamiliar human faces were designed in reference to the pigeon studies [26–28]. Unlike the pigeon experiments, where the test and control group were housed separately, the kea live in a single group. To overcome this shared experience with all ‘familiar’ stimuli, in the study at hand, we introduced a pseudo category for the control group that included randomly selected combinations of familiar and unfamiliar human faces. This made it impossible for the control group birds to classify along familiar/unfamiliar lines and achieved a comparable outcome to having two completely separated groups.

### 2.1. Test subjects

Twelve individuals from the kea flock at the research station Haidlhof participated in testing. All individuals were group housed in a flock of 25 birds in an outdoor aviary (52 × 10 × 4 m) with a sand substrate and a variety of enrichment. The kea had access to water ad libitum and food was provided three times a day. All birds had the same contact with humans around the research station and were experienced with touchscreen discrimination tasks. For details on the individuals, see electronic supplementary material, table S1.

#### 2.1.1. Experimental groups

All test subjects received a two-choice discrimination task on a touchscreen with pictures of familiar (f) and unfamiliar (u) human faces. The 12 individual test subjects were split into two experimental groups (test versus control group) consisting of two test (T1 and T2) and two control groups (C1 and C2) of three individuals each, (electronic supplementary material, table S1). Although the original design had foreseen a counterbalanced distribution of individuals according to sex and rearing method, three individuals had to be substituted in the first weeks of training. This had to be done because either they refused to enter the testing compartment or were unwilling to actively engage in the task. As all participation was on a voluntary basis, these three individuals were exchanged with individuals that also met the criteria for involvement. Rewarding of stimuli was counterbalanced across experimental groups, with half of the test group being rewarded for choosing the familiar (T1) and half for choosing the unfamiliar (T2) stimuli. The control group was reinforced for specific sets consisting of a mixture of familiar and unfamiliar stimuli, electronic supplementary material, table S2.

### 2.2. General procedure

The entire experiment was carried out using the same touchscreen apparatus as that in [37] and others. Each trial consisted of the simultaneous presentation of two pictures of human faces, one on the left and one on the right-hand side of the screen—one rewarded (S+) and one unrewarded (S−) [44]. The kea had to choose between one of the two options by selecting a side. Most kea accomplished this task by touching the screen with either their beak or tongue. S+ selection resulted in a reward tone followed by a food reward in the form of a piece of peanut. S− selection resulted in no-reward tone and no food reward. No correction trials were used. Food rewards were administered via a dispenser wheel mounted to the touchscreen. The experimenter and tested subject were completely visually separated during the touchscreen session and no other birds were allowed into the testing compartment.

The testing compartment, a section of the main aviary, was visually and physically separated from the rest of the aviary by sliding opaque doors. The compartment provided the same general infrastructure (sand substrate, water ad libitum, perches etc.) as the rest of the aviary and could be further divided into two subsections—one waiting compartment and one testing area. The waiting compartment had no visual access to the touchscreen testing area and birds were physically separated by mesh wire. All participation was voluntary.

Each session consisted of 20 trials and each trial contained two stimuli, one shown on the left and one on the right side of the screen, resulting in 40 stimuli per session. A semi-randomized trial order was used to ensure that the positive stimulus did not occur more than three times in a row on the same side of the touchscreen. This was done in order to prevent creating a side bias for the birds and to counter any potential bias they may develop during the course of testing. Each trial and session lasted for as long as it took the individual to complete the task (i.e. a session ended when the individual had completed 20 trials). However, if an individual did not engage with the touchscreen for 20 min (at any time) the session was cancelled. On average there were two sessions per day, per individual, one in the late morning and one in the early afternoon, always post-feeding time.

### 2.3. Stimuli

Coloured photographs of six familiar (f) and six unfamiliar (u) human faces from different angles and heights were used as stimuli. Familiar people in this context were all people who had been in close contact with the kea three to six times per week over the past 5 years. Unfamiliar people were all people that the kea had never seen before. Several steps were taken to ensure that the test subjects could not use other background information in order to solve the task. When taking the pictures that were used as stimuli, each human was placed in front of a white background and had a white sheet draped around their torso and neck. All participants were asked to keep their facial expressions as neutral as possible (no grinning, frowning, laughing) and tie their hair up or move it out of their face. All pictures were corrected for background colour and size and were taken with the same camera (Sony DSC RX100 III).

**Figure 1.**
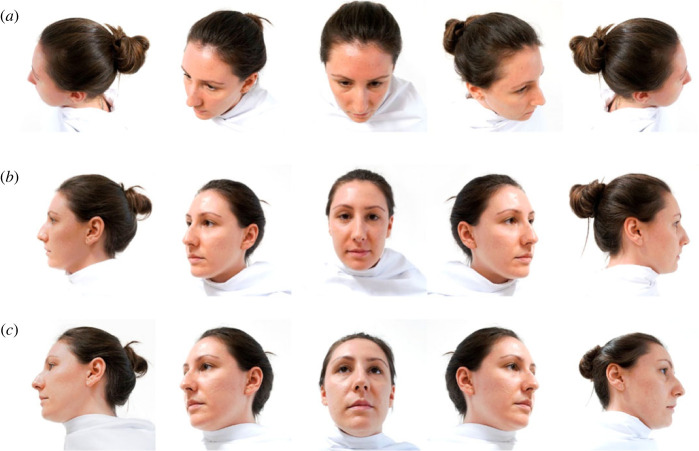
Example full set of stimuli for unfamiliar human A (uA). Face depicted at three different heights: (*a*) above eye level (top), (*b*) at eye level (horizontally) and (*c*) below eye level (below). All heights depicted at five different angles: 0° full right turn, 45° half right turn, 90° full frontal, 135° half left turn and 180° full left turn.

As there were more male familiar people available than female, more male unfamiliar people had to be used to ensure equal numbers. All faces were photographed at three different heights; above eye level, at eye level and below eye level (hereafter top, horizontally and below) and five angles; 0° full right turn, 45° half right turn, 90° full frontal, 135° half left turn and 180° full left turn clockwise from right to left (figure 1). The height/angle combinations along with the different human faces (f and u) made up the testing stimuli. Each familiar and unfamiliar human received a letter from A–F, and in order to differentiate between familiar and unfamiliar, each letter was denoted with the respective abbreviation (f or u). For example, familiar person A was denoted as fA, whereas unfamiliar person A was denoted as uA; familiar person B was fB, and unfamiliar person B was uB and so on.

### 2.4. Conditions

In order to check if kea are able to discriminate between familiar and unfamiliar human faces on the touchscreen the study comprised four conditions: training, generalization, familiarity and reversal. Each session (within the testing condition) had the same amount of stimuli, 40 pictures of the different height/angle combinations, but not the same amount of people. To exemplify, conditions one and two used four different people (two familiar and two unfamiliar) whereas conditions three and four used altogether eight different people (four familiar and four unfamiliar). In order to obtain the same number of stimuli per session in total (namely 40) fewer height/angle combinations had to be used in those conditions that had more people in them. Altogether 92 different stimuli were used across the different conditions. For space reasons, only an example of one full set is given in this section (figure 1), for the extended list of stimuli see the supplementary materials. Each condition was accompanied with its own hypotheses and predictions (table 1).

**Table 1.** Individual conditions with the respective hypotheses and predictions.

conditions	hypotheses		predictions
training	H1	All experimental groups will acquire the discrimination task on the touchscreen.	P1 Kea will be able to learn the discrimination task, regardless of experimental group.
	H2	Identification of conceptual familiarity will have an effect on the speed of the task acquisition for the different experimental groups.	P2.1 If the test groups (T1 and T2) can detect the discriminating factor between the stimuli, namely familiarity, they will take fewer sessions to complete the training condition than the control group (C1 and C2).
			P2.2 If the test groups (T1 and T2) does not recognize the rewarding along familiarity lines early in training, sessions to reach criterion will be similar across experimental groups, as the task is identical without the extra factor of familiarity.
generalization	H3	Kea are able to generalize to novel viewing perspectives.	P3: All test subjects (regardless of experimental group) will be able to generalize from old viewing perspectives to novel viewing perspectives of their respective familiar and unfamiliar human faces.
	H4	The first two sessions of the new condition give indication of task expertise.	P4: If the task was understood already in the training condition then correct selection of old and new stimuli should be high from the onset of testing the new condition (apparent in sessions one and two).
familiarity	H5	Kea apply conceptual familiarity to differentiate between familiar and unfamiliar human faces.	P5: The test groups (T1 and T2) will outperform the control groups (C1 and C2) in the familiarity condition, taking fewer sessions to complete the task, as they can apply the concept of familiarity to the novel stimuli.
	H6	The first two sessions of the new condition give indication of task expertise.	P6: If the concept of familiarity is being applied by the test groups (T1 and T2) then correct selection of old and new stimuli should be high from the onset of testing the new condition (sessions one and two).
reversal	H7	Introducing a partial reversal will illustrate that conceptual familiarity has been applied by the test group.	P7.1: Due to the increased difficulty level for the test groups (T1 and T2) the control groups (C1 and C2) will outperform the test groups (T1 and T2) in the partial reversal task—leading to fewer sessions taken to complete the task.
			P7.2: The partial reversal will negatively affect the performance of the test groups (T1 and T2) on the new stimuli whereas it will remain at a constant level for all stimuli for the control groups (C1 and C2).

#### 2.4.1. Training condition

In the training condition, each kea was acquainted with the two-choice discrimination task and reward contingencies. Four different human faces were used in this condition (*n* = 4; fA, fB, uA, uB). Altogether 10 different height/angle combinations (from the top: 0°, 45°, 90°; horizontally: 0°, 45°, 90°, 180°; from below: 0°, 45°, 90°) were used per human resulting in 40 pictures altogether. For details about reinforcement (S+ and S−) see table S3 of the electronic supplementary material. Criteria for advancement was at least 18 out of 20 correct in three consecutive sessions. We predicted that all individuals will acquire the discrimination task and that the concept of familiarity could have an effect on the speed of the task acquisition for the different experimental groups.

#### 2.4.2. Generalization condition

To test whether kea were indeed differentiating between individuals rather than learning training stimuli by heart, a generalization condition was introduced. In this condition generalization across viewing perspectives was tested. All kea received two novel height/angle combinations of the same human faces (fA, fB, uA, uB) while two old pairings were removed (the new set of height/angle combinations was: 0°, 45°, 135° from the top; 0°, 45°**,** 90°, 180° horizontally and; 0°, 90°, 180° from below) resulting in 40 stimuli altogether. For details on reinforcement (S+) see electronic supplementary material, table S4. Criteria for advancement was at least 18 out of 20 correct in three consecutive sessions, and within those at least one session 100% correct on the new stimuli and no session with more than one novel stimulus trial incorrect.

In the generalization condition all experimental groups were faced with the same task of applying the expertise they had acquired in the training condition to novel perspectives of the same human faces. Therefore, in this condition the understanding of the task, namely correctly identifying the respective S+ stimuli, was tested rather than the application of the concept of familiarity. We predicted that all individuals, regardless of experimental group, will be able to generalize from old viewing perspectives to novel viewing perspectives of their respective familiar and unfamiliar human faces.

#### 2.4.3. Familiarity condition

The familiarity condition was the critical test investigating whether the test groups (T1 and T2) were applying the concept of familiarity. To test for categorization along lines of familiarity, four novel human faces (fC, fD, uC, uD) were added to the already existing ones (fA, fB; uA, uB), with five height/angle combinations for all faces (45° from the top; 90°, 180° horizontally; and 45°, 90° from below) to result in 40 stimuli altogether as before, electronic supplementary material, table S5. In order to prevent inference by exclusion, pictures were only paired with pictures of the same novelty. That is, in each single session, previously used human faces (fA, fB; uA, uB) were paired, and novel human faces (fC, fD; uC, uD) were paired. This resulted in two sets of trials per session: 10 trials of old pairings and 10 trials of novel pairings. The old and novel pairings were randomized in order, resulting in a mixed session of old and novel picture trials. Criteria for advancement was at least 18 out of 20 correct in three consecutive sessions, with no less than 9 out of 10 correct in the novel stimuli trials in those sessions.

If the test groups (T1 and T2) recognize the distinguishing factor between their stimuli, hence applying a concept of familiarity to categorize the S+ and S− and thus solve the task, it should become apparent in this condition. The distinct difference between the experimental groups in this condition being that the test groups (T1 and T2) had the advantage that they could use the concept of familiarity to inform their choices. The control groups (C1 and C2), in turn, did not have this possibility, as they are being reinforced on randomly mixed sets of familiar and unfamiliar faces. Hence, the test groups would have the information to choose the S+ immediately, while the control groups would need to learn the reward contingencies for the novel stimuli pairs. We hypothesized that kea will apply conceptual familiarity to differentiate between familiar and unfamiliar human faces. We therefore predicted that the test groups (T1 and T2) will outperform the control groups (C1 and C2) in the familiarity condition as they can apply the concept of familiarity to the novel stimuli. We also assumed that the first two sessions of the new condition would give the strongest indication of task expertise and predicted that if the concept of familiarity is being applied by the test groups (T1 and T2) then correct selection of old and new stimuli should be high from the onset of testing the new condition (sessions one and two).

#### 2.4.4. Reversal condition

In the final step a partial reversal condition was introduced. In this condition the test group birds were being hindered in their stimulus response by introducing reversed reinforcement for novel stimuli presented in half of the trials. This additional level of complexity helps verify how strongly the test group adheres to the categorization according to familiarity. Half of the trials (10) used the human faces from training (fA, fB, uA and uB) and half (10) used novel human faces (fE, fF, uE, uF), electronic supplementary material, table S6. Five pictures per human from different height/angle combinations (from the top: 0°, 45°; horizontally: 135°, 180°; and from below: 90°) were used in order to obtain 40 stimuli altogether. As in the familiarity condition, stimuli were paired according to novelty in order to avoid categorization on the basis of exclusion. By contrast to all other conditions, the reinforcement for the test groups (T1 and T2) with regard to familiarity was reversed for the novel stimuli. The partial reversal was only relevant for the test groups (T1 and T2), with the novel pairings being reinforced counter to the last three conditions, as rewarding for the control groups (C1 and C2) consisted of random sets of familiar and unfamiliar human faces, thus no consistency had been provided that could be reversed. Their task therefore remained the same, discriminating between random mixed sets of familiar and unfamiliar human faces. Criteria for completion (as in the previous condition) was at least 18 out of 20 correct in three consecutive sessions, with no less than 9 out of 10 in the novel stimuli trials in those sessions.

Note that the test groups were faced with two contradicting reward contingencies within one session, with half the stimuli in the session reinforced as per usual and half with reversed reinforcement. Hence, we predicted that, due to the increased difficulty level for the test groups (T1 and T2), the control groups (C1 and C2) will outperform the test groups (T1 and T2) in this condition. More specifically, we predicted that the partial reversal will negatively affect the performance of the test group (T1 and T2) with the new stimuli, whereas it will remain at a constant level for all stimuli for the control group (C1 and C2).

All conditions were tested in direct succession on the basis of completion. Once a test subject completed all four conditions it was removed from the test.

### 2.5. Data analysis

Initial data analysis was performed with IBM SPSS 25.0.0.0 (IBM Corp 2017). Each parameter of interest was tested for normal distribution with the Shapiro–Wilk Test. The Mann–Whitney *U*-test was used to check for significant differences between the experimental groups in completing the task. Total session numbers per condition and individual were compared. Additionally, sessions one and two of all but the training condition were analysed separately using the Mann–Whitney *U*-test to check for any effects that could be present at the onset of testing a new condition (i.e. adding new stimuli). The *α*-level was set at 0.05 and all tests were performed at exact level [45,46]. We are aware that the samples compared comprise only few individuals and hence the power of these comparisons will be low. However, even for the smallest sample comprising three and four individuals in the two groups to be compared, an exact Mann–Whitney *U* test can still reveal a trend (two-tailed *p* = 0.057) and for the others comprising five and six individuals it can reveal a *p* = 0.004. In addition, we also fitted several models which make use of the much more detailed data we obtained with the experiments (see below).

To estimate the extent to which the probability of choosing the correct stimulus changed over the course of sessions and trials within sessions and how this change differed between the two groups (test and control) we fitted a series of three generalized linear mixed models (GLMM; [47]) with binomial error structure and logit link function in R [48]. With model 1 we analysed the familiarity condition, and with models 2 and 3 we analysed the keas' choices when confronted with familiar and novel stimuli, respectively, in the partial reversal condition. All models had the same fixed effects structure.

We expected learning to take place in all experiments and it seemed plausible to assume that the probability of choosing the correct stimulus would increase across sessions but also across trials within sessions, and this latter effect could be particularly pronounced in intermediate sessions (electronic supplementary material, figure S15). Furthermore, we expected learning to be easier and hence steeper in the test as compared with the control group. Hence, we included in all models the two three-way interactions between group, trial number, and session as well as session squared, respectively.

As it seemed likely that variation in the orientation of the two pictures affects the probability of a correct choice, we further included the horizontal viewing angles of the two pictures (numeric covariates) and also the vertical viewing angles (heights) of the two pictures (factors indicating whether the view was frontal or not) as further fixed effects. Hence, the structure of all models with regard to the fixed effects was correct choice (no/yes) ∼ group * trial.nr * (session.nr + .nr^2^) + horizontal angle of the correct picture + vertical angle of the correct picture + horizontal angle of the incorrect picture + vertical angle of the incorrect picture. Additionally, each model comprised several random intercepts and random slopes effects which are detailed in the electronic supplementary material, information.

For all models we conducted a full-null model comparison. Such a full-null model comparison aims to avoid ‘cryptic multiple testing’ and keeps type I error rate at the nominal level of 0.05 also in the presence of multiple terms to be tested [49]. To this end, we compared each full model with a respective null model that lacked group and all interactions with it in the fixed effects part but was otherwise identical to the full model. If such a comparison reveals significance, it indicates group differences in the overall number of correct choices and/or steepness of learning across and/or within sessions.

We fitted the models in R (v. 3.6.6; [50]) using the function glmer of the package lme4 (v. 1.1–21; [51]). Prior to fitting the models we z-transformed trial number, session number and the two horizontal viewing angles to a mean of zero and a standard deviation of one to ease model convergence and achieve easier interpretable estimates [52]. Before including factors as random slopes, we manually dummy coded and then centred them. For further specifications see the supplementary material.

## 3. Results

In summary, 1059 sessions in total were tested over a nine-month period. Eleven of the twelve birds acquired the discrimination task in training. One bird from the control group (Papu) did not manage to reach criterion within the testing period. All 11 birds were able to generalize from their respective S+ and S− humans to new perspectives of the same humans. Seven individuals (four from the test group and three from the control group) successfully completed the familiarity condition. Four birds, two from the test and two from the control group successfully completed the final condition, reversal. See table 2 for details on the individuals, experimental group, sex, age and overview of total number of sessions per individual and condition.

**Table 2.** Overview total number of sessions per individual and condition separated into the experimental groups (test versus control group).^a^

experimental group	individual	sex	age	training	generalization	familiarity	reversal
test group 1	John	♂	18	21	9	86	22
test group 1	Kermit	♂	13	31	32	(58)	
test group 1	Plume	♀	13	23	4	61	
test group 2	Coco	♀	10	23	4	57	
test group 2	Pick	♂	13	85	11	(25)	
test group 2	Roku	♂	9	11	6	12	55
control group 1	Elvira	♀	17	107	8	(6)	
control group 1	Lilly	♀	10	64	6	31	
control group 1	Paul	♂	7	29	3	30	16
control group 2	Frowin	♂	13	27	3	80	23
control group 2	Papu	♀	14	(121)			
control group 2	Sunny	♀	10	70	9	(54)	

^a^Missing values in the condition columns denote that the individual did not participate in this condition. Numbers in brackets mark individuals that started but did not complete the condition.

### 3.1. Training condition

A total of 11 test subjects successfully completed training. One bird from the control group C2 (Papu) did not manage to reach criterion in training (at least 18 out of 20 correct in three consecutive trials) and was therefore excluded from the analysis. On average test subjects spent 44.6 sessions in the training condition (test group (median: 23 (interquartile range: 26), *n* *=* 6; control group: 64 (61), *n* *=* 5) with a huge variation in the data. There was no significant difference (Mann–Whitney *U*-test: *U* *=* 6.000, *n*_test group_ = 6, *n*_control group_ = 5, *p* = 0.126) between the mean number of sessions each experimental group (test versus control) took to complete the training condition. Although the control group on average spent slightly more sessions in training.

### 3.2. Generalization condition

All 11 test subjects that completed the training condition also completed the generalization condition successfully. Furthermore, with the exception of one outlier, all individuals performed at an equal level in the generalization task. There was no significant difference (*U* *=* 21.000, *n*_test group_ = 6, *n*_control group_ = 5, *p* = 0.329) between the experimental groups in solving the generalization task. However, looking at the first two sessions in the new condition (generalization) illustrated that initially there was a significant difference between the experimental groups in solving the task. If we look at session one of the generalization condition, we can see that there was a significant difference between the groups (*U* *=* 2.500, *n*_test group_ = 6, *n*_control group_ = 5, *p* = 0.017). However, this was no longer the case in session two (*U* *=* 14.000, *n*_test group_ = 6, *n*_control group_ = 5, *p* = 0.931). Interestingly, the control groups (C1 and C2) outperformed the test groups (T1 and T2) in the generalization task by obtaining higher scores in the first session.

### 3.3. Familiarity condition

The familiarity condition investigated whether the test groups (T1 and T2) were able to categorize human faces on the basis of familiarity and hence take fewer sessions to reach the advancement criterion. However, the results indicate that there was no significant difference between the mean number of sessions to complete the familiarity condition per experimental group (*U* *=* 7.000, *n*_test group_ = 4, *n*_control group_ = 3, *p* = 1.00). Only seven individuals altogether completed the familiarity task, four from the test (T1 and T2) and three from the control groups (C1 and C2). With regard to the familiarity model (model 1), we did not find a significant full-null model comparison (*X^2^* = 4.580, d.f. = 6, *p* = 0.599). The model revealed that both the test and the control group learned to choose the correct stimulus at about the same pace (electronic supplementary material, figure S11).

**Figure 2.**
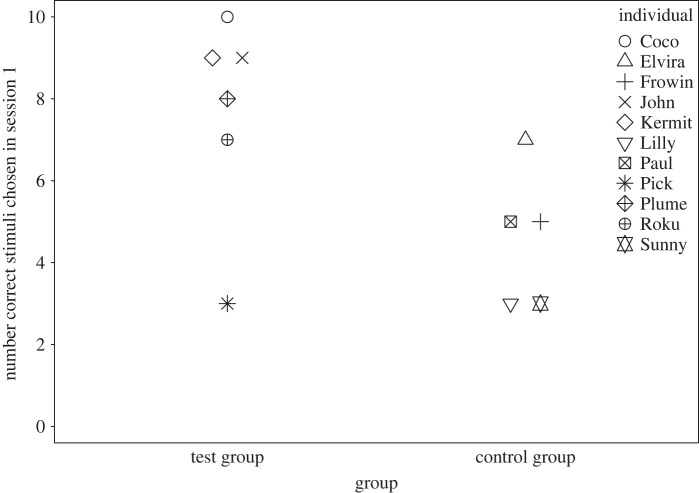
Number of correct new stimuli in session one of the familiarity condition, separately per experimental group (*n* = 11, Mann–Whitney *U*-test: *U* = 25.500, *p* = 0.052).

When considering the first session of the new condition (familiarity), however, there was a marginally non-significant difference between the experimental groups in session one (*U* *=* 25.500, *n*_test group_ = 6, *n*_control group_ = 5, *p* = 0.052); here we considered all individuals that completed the session, not only those that completed the condition. The test group (T1 and T2) performed significantly better on average than the control group (C1 and C2; session one, figure 2). With the test group having up to 100% success rate in session one and up to 95% in session two.

Looking closer at this result, the significant difference in performance between the two experimental groups was due to the new stimuli in session one of the familiarity condition (*U* *=* 25.500, *n*_test group_ = 6, *n*_control group_ = 5, *p* = 0.052). The test group on average had 7.7/10 correct on the new stimuli (median: 8.5 (6), *n* *=* 6) with one individual getting 10/10 correct in the first session. Whereas the control group on average had 4.6/10 correct on the new stimuli (median: 5(3), *n* *=* 5), with no individual achieving more than 7/10 correct in the first session. Five individuals of the test group chose correctly in the first trial the new stimulus appeared (*n* *=* 6), whereas only two individuals of the control group managed to identify the correct new stimulus in the first instance (*n* = 5). Both groups performed similarly well in correctly selecting the old stimulus (Mann-*U* *=* 14.000, *n*_test group_ = 6, *n*_control group_ = 5, *p* = 0.93). The second session already showed no more significant difference (*U* *=* 22.400, *n*_test group_ = 6, *n*_control group_ = 5, *p* = 0.49) between the experimental groups concerning the selection of new stimuli.

Importantly, there was no difference between the experimental groups’ performance on the old stimuli in either session one or two. This indicates that overall the test group (T1 and T2) performed better than the control group (C1 and C2) in the first session of the familiarity condition by having more success in correctly identifying the new stimuli in the first session, although this effect was already lost in the second session.

### 3.4. Condition reversal

Only four of the initial seven individuals that entered the condition managed to complete the reversal task, two from the test (T1 and T2) and two from the control group (C1 and C2). There was no difference between the experimental groups in the reversal condition. The model did not find significant differences between the two experimental groups, neither with regard to old stimuli (model 2; full-null model comparison: *X^2^* = 7.978, d.f. = 6, *p* = 0.240) nor with regard to novel stimuli (model 3; *X^2^* = 10.296, d.f. = 6, *p* = 0.113). The model revealed that both the test and the control group learned to choose the correct stimulus at about the same pace (electronic supplementary material, figure S12). In fact, the probability to choose the correct old stimulus was high from the first session on, and this was the case in both the test and the control group (electronic supplementary material, figure S13). Of the four individuals, Roku from the test group and Paul from the control group completed the first session with 10/10 correct on the old stimuli. Test group individual John selected 9/10 correct and control group individual Frowin 8/10 correct on the old stimuli (electronic supplementary material, figure S18). If we look further into testing, by session eight John achieved 19/20 correct with the incorrect choice being on an old stimulus and therefore reaching the criterion for the first time. All individuals fluctuated in their performance across sessions with Roku meeting criterion once by session 25, Paul by session 14 and Frowin by session 18.

The probability to correctly choose a novel stimulus was at about chance level in the beginning of the first session in both groups and gradually increased at about the same pace in the control as well as the test group (electronic supplementary material, figure S13). All four individuals scored around the 50% mark concerning the new stimuli. Importantly, test group individual John achieved 7/10 correct choices on the new stimuli by session two.

## 4. Discussion

The present results illustrate that kea can learn to discriminate between familiar and unfamiliar human faces on the basis of two-dimensional information, which has never been shown in parrots before. There is a strong indication that the kea applied the concept of familiarity to solve the task, as the results from training and familiarity (session one) suggest.

Although the test group individuals spent less time on average in the training phase when compared with the control group, there was no significant difference between the groups. Similar to the 2013 pigeon studies (Stephan *et al.* [28]) this suggests that the task was equally demanding for all groups. However, only five out of the six control group subjects managed to acquire the task, whereas all of the test group subjects (*n* = 6) succeeded in learning the task. Additionally, if the performance above chance level (16 out of 20 in two consecutive trials) was considered, the test group outperformed the control group significantly. This suggests that the test group may have already detected the concept of familiarity in the training phase. Once the test group identified the distinguishing characteristic (familiar versus unfamiliar) they were able to apply this concept to the task, making it easier for them to solve it. By contrast, the control group had to learn specific reward contingencies for each individual human face and did not have the opportunity to identify any concept. The results could be an indication that, although performance was significantly above chance level, a certain level of expertise was necessary to complete the task. Being able to perform above chance level, in this regard, is quite different to having acquired a level of expertise required to achieve the criterion set for advancement, the latter of which was chosen to allow the subject to apply this knowledge in the next step, when generalizing to new stimuli was required. The results therefore suggest that while acquiring the task was facilitated by the familiarity categorization, achieving the level of expertise we aimed for (namely at least 18 out of 20 correct in three consecutive sessions) needed additional practice, which allowed the control group to catch up in their performance.

As predicted, the generalization condition showed no difference between the experimental groups. Both experimental groups had been acquainted with their task in training and were asked to transfer their knowledge to new viewing perspectives of the same human faces. However, interestingly, a closer analysis of the data illustrated that within the first session of the generalization condition, there was a significant difference between the groups. The first session of a new condition shows the spontaneous reaction based only on knowledge from the prior condition rather than any further information acquired in the new condition. Therefore, a high performance in the first session of a new condition indicates knowledge of the task. Surprisingly, the difference was in favour of the control group, which outperformed the test group in this first attempt at the task. The control group did spend more time in training than the test group, albeit not significantly more sessions. Nonetheless, the result may be a simple training effect of more practice leading to better performance. Alternatively, a similar process as illustrated by Narula *et al*. [53], concerning the generalization abilities of zebra finches (*Taeniopygia guttata*), may have been affecting the outcome. Narula and colleagues found that birds, which learned an initial task through trial and error, were faster at solving a subsequent generalization task. By contrast, those birds that used ‘strongly regularized classifiers' [53, p. 8] were fast learners in the initial training phase but slower to generalize.

The familiarity condition investigated whether the test group was able to apply a concept of familiarity to solve the discrimination task. Four out of six test group and three out of six control group birds successfully completed the familiarity condition. As predicted, the first session of the familiarity condition illustrated that the concept of familiarity was indeed applied by the test group. In this condition, we presented four novel human faces in addition to the already existing ones. The test group had significantly more correct choices, in session one, than the control group. This illustrates that the test group birds must have applied the concept of familiarity to solve the task in the first instance. Furthermore, the actual significance was not only in the performance levels overall, but specifically in correctly identifying the new stimuli (as a reminder, there were 10 novel and 10 old stimuli in each session in the familiarity condition). There was no difference between the experimental groups concerning the old stimuli selection, demonstrating the deciding factor was not the old stimuli but the new stimuli selection. This is a clear indication that the test group was applying the concept of familiarity to solve the new task. Interestingly, this initial advantage did not carry over to the following sessions, with the experimental groups performing at an equal level overall when considering all sessions to completion.

Only four individuals, two from each experimental group, successfully completed the reversal task, suggesting a high difficulty level of the task overall. There was no difference between the experimental groups in this condition, and counter to the hypothesis and predictions, the results did not indicate that individuals from any group could solve the task more quickly. This suggests that all four test subjects learned the new task by heart, applying a trial and error technique overall. Simultaneously, this indicates behavioural flexibility in problem solving especially for the test group. The test group subjects did not only continue to apply the rule they had acquired in the previous conditions for half of the trials in each session (10/20) but additionally acquired a reversal for the other half of their trials (10/20). They were able to flexibly adjust their behaviour for two contradictory tasks within each session, which is quite a remarkable result. One test group individual (John) in particular illustrated a high proficiency in behavioural flexibility and a steep learning curve with a successful task acquisition by session eight. This high proficiency in flexibly adapting to novel stimuli would also be in line with the ecological demands kea face in the wild, with complex social structures and unpredictable resources [54–56].

Several aspects of the study may have had implications on the overall results of this study. One aspect that is important to consider when comparing the experimental groups is that not only were there differences in the performance between the test and control groups, but also between individuals within groups. Several factors may be relevant when looking at the individual performance of the test subjects. There may have been slight variation in the duration, intensity, and frequency of interactions with humans even between individuals of a single flock. Individuals that are shyer or less interested in heterospecific interactions may also have less experience and hence familiarity with the humans used in the study. Alternatively, some of the humans may have interacted with the birds more and with a different quality (e.g. valence) than others. Such differences were observed by Stephan *et al*. [28] in their pigeon studies on object discrimination. They suggest that there may be ‘differences in the frequency of interaction or the relevance of the familiar object for different subjects' [28, p. 991] and that potentially physical interaction may be necessary to facilitate the application of abstract familiarity. A similar process was illustrated by Carducci and colleagues who found that ‘touch accelerated learning speed for visual object recognition’ in kea and capuchin monkeys (*Sapajus* spp.) [43, p. 199]. They suggested considering that the haptic domain may be especially relevant for those species that regularly manipulate objects in their natural environment. It would, therefore, be important for future studies to consider not only the frequency of interactions but to also reflect on their intensity, duration and nature (physical interaction versus non-physical).

All individuals had prior experience with the touchscreen and participated in object discrimination [37] and/or reversal learning [57] tasks. While exposure to those previous tasks might have facilitated learning speed in the task at hand it is unlikely that it had a decided positive effect overall, as the Laschober study found that there was considerable variation between and within individuals [57] when solving the reversal task. Furthermore, the 2021 study applied a standardized approach with all individuals receiving 60 mid-session reversal sessions whereas the study at hand applied a criterion-based approach allowing each individual to remain in one condition until a preset criterion was met (see Conditions section). Such an approach should reveal if learning set acquisition [58] is potentially at play by the number of trials it takes individuals to complete the tasks. We would expect a gradual increase in proficiency to solve the task, and hence decrease in trials to reach criterion, over consecutive problems/conditions if individuals were reliant on learning set formation [59–61]. However, our results showed clear fluctuation in the performance between conditions/consecutive problems (table 2). We, therefore, do not assume learning set acquisition. We acknowledge that there may have been training effects concerning the potential of a reversal occurring, albeit a partial reversal (two contrary reward contingencies presented simultaneously) never having been tested before. Further comparative analysis of the present study with the reversal task by Laschober *et al*. [57] would be necessary to identify any overlapping effects with full clarity.

Other possible confounding factors could have been (i) the functionality of the touchscreen and (ii) the breeding season. Unfortunately, the touchscreen did not provide reliable reinforcement, with the feeding dispenser clogging on several occasions. This was mainly due to outside conditions, with high humidity causing more clogging than low humidity. The effect of irregular (or intermittent) reinforcement on learning has been illustrated in many studies (e.g. [62]). Additionally, the testing period also covered all seasons including the breeding season. During this time the individuals are busy with pairing, finding nesting sites and displaying territorial displacement. This is a stressful time for all individuals in the flock, with high tension and more frequent agonistic behaviour. Especially lower ranking animals are affected by this situation. Both these factors (touchscreen and breeding season) may have affected some individuals more than others and it would be valuable for future studies to investigate the specific influence on individual performance levels.

A caveat of our study is the sample size, as the number of birds tested was relatively small and further decreased as birds progressed through the sequence of experiments. This raises particularly the question of whether the, in part, non-significant differences between the two groups of birds we found could be due to a lack of power. However, we think that this is not the most likely explanation for two reasons. First, in part we found differences between the two groups, indicating that the relatively small sample sizes did not preclude significant effects *per se*. Second, the fitted models (electronic supplementary material, figure S11 and S12) revealed that the two groups overall performed very similarly. As such, these revealed no obvious hints that there were indeed stronger differences between the two groups. Nevertheless, a replication with a larger sample would be desirable to confirm (or refute) our findings.

An aspect of importance regarding the interpretation of the results from this study, and potentially others using two-dimensional representations of individuals, is the concept of recognition. Being able to discriminate between familiar and unfamiliar heterospecifics is not the same as recognition. Studies relying on a cross-modal design to test heterospecific recognition have illustrated that both vertebrates such as pigs (*Sus domesticus*) [63] and invertebrates such as octopuses (*Enteroctopus dofleini*) [64] have the ability to truly recognize and differentiate between human handlers. These studies, however, did not rely on two-dimensional representations alone to test for this ability. As elaborated in the dog studies on discrimination of familiar human faces [65,66], the two terms should not be confused. Animals rely ‘on different modalities, ranging from chemosensory (…) to acoustic (…) and visual cues' [66, p. 881] to recognize individuals. Using only two-dimensional pictorial representations to test for recognition may be misguided. As Weissman and Spetch postulated, ‘separate evolutionary pathways and distinct differences in existent avian and mammalian visual systems mean that researchers cannot assume that birds see pictures the way humans do’ [67, p. 117]. We therefore cannot clearly discern what information the birds used to solve the task, whether it is shapes, textural information or specific facial cues [68].

Approaches to disentangle what information is being used in face processing include the provision of manipulated pictorial stimuli, such as half faces, mirrored and scrambled/chimeric features. Peirce *et al.* [69], for instance, applied such an approach and illustrated that sheep can clearly differentiate between familiar and unfamiliar human faces using two-dimensional representations alone. However, they found that there are stark differences in processing con- versus hetero-specific faces. While conspecific differentiation relied heavily on a left visual field bias, heterospecific differentiation showed a marginal reverse bias to the right visual field [69, p. 23]. Additionally, Kendrick *et al.* [20] showed that (in the mammalian brain) ‘a small population of cells in the temporal and medial prefrontal cortices (…) encodes faces, as opposed to other visual objects' [20, p. 165]. This can be used to measure cell response to face versus non-face stimuli and together with behavioural responses gives good indication of facial recognition of, in their case, sheep. However, the visual forebrain structure of birds ‘bears almost no resemblance to (….) the mammalian neocortex (…) [which is responsible for terminating] biologically relevant visual precepts (such as faces)’ [70, p. 1]. Therefore, while we have good evidence how mammalian species process visual information in regard to categorical representations and can utilize this knowledge to test for potential recognition, such information is still largely missing for avian species. By testing kea on their ability to differentiate between pictures of familiar and unfamiliar humans, we therefore cannot make any statements about their ability to recognize the individuals we presented to them, just that they probably recognized certain features familiar to them. Further studies, with a focus on selecting only certain features, providing cross-modal information or monitoring brain activity would have to be devised to clarify this aspect.

In conclusion, the results illustrate that kea can learn to discriminate between familiar and unfamiliar heterospecifics on the basis of pictorial two-dimensional representation, supporting their object-to-picture transferral abilities shown by Wein and colleagues [37]. Furthermore, the results suggest that they can apply the concept of familiarity to discriminate between individuals. The results provide novel empirical evidence on abstract categorization capacities in parrots, placing kea's differentiation abilities in line with those of great apes [35] as well as those of pigeons [26]. Finally, the study gave indications that it is possible for kea to acquire a partial reversal, which requires the continued application of a learned categorization while simultaneously acquiring a reversed reward contingency within a session. Such a capacity is clearly very difficult, as only a restricted subset of individuals managed to accomplish this feat, future studies will have to investigate this capacity further.

## Data Availability

Prep data, R scripts and R workspace are available from ScienceDB repository: https://doi.org/10.57760/sciencedb.07488 [71]. Additional information is provided in electronic supplementary material [72].
